# Factors Determining Not Returning to Full-Time Work 12 Months After Mild Ischemic Stroke

**DOI:** 10.1016/j.arrct.2022.100245

**Published:** 2022-11-12

**Authors:** Georgios Vlachos, Hege Ihle-Hansen, Torgeir Bruun Wyller, Anne Brækhus, Margrete Mangset, Charlotta Hamre, Brynjar Fure

**Affiliations:** a Department of Geriatric Medicine, Oslo University Hospital, Oslo, Norway; b Department of Neurology, Oslo University Hospital, Oslo, Norway; c Institute of Clinical Medicine, University of Oslo, Oslo, Norway; d Department of Internal Medicine, Bærum Hospital, Vestre Viken, Norway; e Department of Physiotherapy, Oslo University Hospital, Oslo, Norway; f Department of Internal Medicine, Central Hospital, Karlstad, Region Värmland, Sweden; g School of Medical Sciences, Örebro University, Sweden

**Keywords:** Stroke, Work, Association

## Abstract

**Objective:**

To evaluate prevalence and factors determining not returning to full-time work 1 year after first-ever mild ischemic stroke.

**Design:**

Prospective, observational cohort study with 12-month follow-up.

**Setting:**

Stroke units and outpatient clinics at 2 Norwegian hospitals.

**Participants:**

We included 84 (N=84) full-time working, cognitively healthy patients aged 70 years or younger who suffered an acute first-ever mild ischemic stroke, defined as National Institutes of Health Stroke Scale (NIHSS) score ≤3 points.

**Interventions:**

Not applicable.

**Main Outcome Measures:**

Vascular risk factors, sociodemographic factors, stroke localization, and etiology were recorded at inclusion. Cognitive impairment, anxiety, depression, fatigue, and apathy 12 months after stroke were assessed with validated instruments. Logistic regression analyses were performed to find correlates of not returning to full-time employment.

**Results:**

Of 78 patients assessed 1 year after stroke, 63 (81%) had returned to work, 47 (60%) to full-time employment status. Modified Rankin scale score >1 (adjusted odds ratio, 12.44 [95% confidence interval, 2.37-65.43], *P*=.003) at follow-up was significantly associated, and diabetes (adjusted odds ratio, 10.56 [95% confidence interval, 0.98-113.47], *P*=.052) was borderline significantly associated with not returning to full-time work. Female sex, NIHSS at discharge, anxiety per point on the anxiety scale, depression per point on the depression scale, and fatigue per point on the fatigue scale were significantly associated with not returning to full-time work after 1 year, but these associations were only seen in the unadjusted models.

**Conclusions:**

Low functional level that persists 12 months after stroke is related to not returning to full-time work. Patients with diabetes mellitus, female patients, and patients with a higher score on fatigue, anxiety, and depression scales may also be at risk of not returning to full-time work post stroke. Working patients should be followed up with a particular focus on factors determining participation in work and social life.

## Highlights

• A total of 40% of the patients did not return to full-time work 12 months after a first-ever mild ischemic stroke.
• Low functional level expressed as modified Rankin scale score >1 at 12-month follow-up was significantly associated with not returning to full-time work.
• Diabetes mellitus was a borderline significant factor of not returning to full-time work.

Unemployment is associated with poor physical health^1^ and reduced quality of life.^2^ For some patients with stroke, work can be a cause of stress and therefore potentially a risk, whereas for others it is a way of demonstrating recovery.^3^ It has previously been found that 53% of patients returned to work 1 year after stroke,^4^ with the proportion being lower in women than in men.^5^ Another study^6^ indicated that 47% of patients working fulltime prestroke were still working fulltime 1 year later, while 27% were no longer in work, and 24% were working fewer hours a week. Predictors of returning to work were less severe neurologic deficits, better cognitive ability, greater independency in daily life activities, age younger than 51 years, and male sex.^4^^,^^7^ Psychiatric morbidity, depressive symptoms, and poststroke fatigue might hinder younger patients from returning to professional activity for as long as 2 years after first-ever mild stroke.^6^^,^^8^, ^9^, ^10^

Functional outcome after stroke has been improved during the last years because of both intravenous thrombolysis and mechanical thrombectomy.^11^, ^12^, ^13^ Thrombolysis for first-ever ischemic stroke was independently associated with a reduced rate of dementia in one study.^14^ The relationship between thrombolytic treatment and return to paid work has not been much investigated,^15^ but a recent article^16^ reported a high proportion of patients treated with thrombolysis returning to work (64%) 1 year after stroke. Age 41-60 years, high stroke severity expressed as National Institutes of Health Stroke Scale (NIHSS)^17^ score ≥5, and female sex were associated with lower odds of workforce attachment.^16^ The effect of endovascular stroke treatment on work ability is largely unknown.

One incentive to initiate this study was our own experience from the stroke outpatient clinic, where we observed that several young working patients with minimal or no neurologic sequelae after a mild stroke reported difficulties involving cognitive functions. In addition, some patients struggled with fatigue, indifference or apathy, anxiety, depression, or mood swings. It was previously shown that hidden outcomes can hinder the patients from returning to normal daily activities and affect both family and work life.^18^ Unemployment is often associated with poorer physical^1^ and mental health^19^ and reduced quality of life.^2^ In a Dutch study, one-third of the participants who had not gone back to work 1 year after a mild-to-moderate stroke were unsatisfied with their occupational situation.^6^ In addition, the work satisfaction of working patients increased with the daily number of hours spent in a paid work.

Most studies about returning to work after stroke have included patients with mild-to-moderate ischemic and hemorrhagic strokes defined as NIHSS score ≤15,^20^ while our study is one of the few studies that has included patients with only mild ischemic strokes defined as NIHSS score ≤3. Other authors^21^ have found that stroke size, localization, and etiology, i.e. large anterior strokes, and stroke caused by large artery atherosclerosis and cardioembolism, are associated with not returning to work 1 year after ischemic stroke. We previously^22^ found that younger age at stroke onset and finding of multiple cerebral infarctions were associated with cognitive impairment 1 year after stroke.

Overall, the aim of this prospective cohort study was to identify the prevalence of not returning to full-time work and to study whether factors such as demographic characteristics, stroke etiology, localization, and reperfusion therapy were associated with not returning to full-time work 12 months after first-ever mild stroke in patients 70 years or younger.

## Methods

### Population

The included patients were admitted with an acute stroke to the stroke units at Oslo University Hospital, Norway and Bærum Hospital, Norway in the period from December 2014 until December 2016. Details regarding the study design have previously been published.^23^ In this substudy, we included patients employed fulltime, prescribed by law in Norway as working at least 37.5 hours a week, at stroke onset. Inclusion criteria were age 18-70 years and that the participants had a mild first-ever ischemic stroke defined as a NIHSS^17^ score ≤3 points at discharge.^24^

Patients who did not speak Norwegian, patients with diagnosed dementia or cognitive impairment defined as a score > 3.2 on the short form of the Informant Questionnaire on Cognitive Decline in the Elderly,^25^ and patients with known psychiatric disease were not eligible. Patients who had a new stroke during the first year after stroke were not invited to follow-up.

### Assessments

We recorded age, sex, educational level in years, and marital state at stroke onset in addition to vascular risk factors as treated hypertension, hyperlipidemia, coronary heart disease, atrial fibrillation, cigarette smoking, diabetes mellitus, body mass index, and presence of any apolipoprotein E-epsilon 4 alleles.

The diagnosis of ischemic stroke was based on the medical history, findings on the neurologic examination, and findings of an acute infarction on cerebral computed tomography (CT) or magnetic resonance imaging (MRI) scans. Patients presenting with neurologic deficits lasting more than 24 hours, but with no acute lesions on CT or MRI, were also considered to have an ischemic stroke.^26^

The ischemic strokes were classified by etiology according to the Trial of ORG 10172 in Acute Stroke Treatment (TOAST) classification.^27^ The Barthel Index score and the modified Rankin scale (mRS) score^28^^,^^29^ was used to assess the general functional level at discharge and at 12-month follow-up.

### Twelve-month follow-up

Study participants were invited to follow-up in the outpatient clinic. The cognitive and emotional assessments were performed by either a stroke physician, an occupational therapist, or a research nurse. These professionals had been trained by experienced physicians with high competence and interest in the field.

### Cognitive and emotional assessments

Cognitive and emotional functions were evaluated using an extended battery of screening tests.^23^

The Mini-Mental Status Examination Norwegian Revision 2 (MMSE-NR2) is considered to be a screening test of global cognitive functioning.^30^ To examine particular cognitive domains, we used the Clock Drawing Test^31^ (executive and visuospatial function), the Trail Making Test (TMT) A^32^ (focused attention, psychomotor speed) and B (divided attention, executive function, psychomotor speed), the Controlled Oral World Association (COWA) Verbal Fluency Test^33^ (attention, executive function, psychomotor speed, language ability), the Rey-Osterrieth Complex Figure Test (ROCF)^34^ (executive and visuospatial function, memory), and the California Verbal Learning Test II (CVLT II)^35^ (learning ability, memory). Because of the participants’ high educational level and low mean age (52.4 years), we chose a higher score (<27/30 points)^36^ than usual as a cutoff for the MMSE-NR2.^30^ For the Clock Drawing Test, the standard cutoff of 4 of 5 points was used,^31^ while the correct administrations within 60 seconds for the TMT A and 120 seconds for the TMT B were chosen as cutoffs.^32^

Because of the sparse evidence for differences related to age and sex on CVLT II scores, to age on ROCF, and to sex and education level on COWA and Trail Making Tests, raw scores were converted into standardized scores (mean±SD, 50±10) based on appropriate normative sample according to test manuals used by the Norwegian Registry of Persons Assessed for Cognitive Symptoms in Specialist Health Care services. In accordance with the criteria of the International Society for Vascular Behavioral and Cognitive Disorders,^37^ mild cognitive impairment can be present when the performance on validated cognitive tests in 1 or more of the cognitive domains of attention, processing speed, executive function, learning and memory, language, visuoconstructional-perceptual ability, praxis-gnosis-body schema, and social cognition is 1-2 SD below appropriate norms. Accordingly, we defined a score of at least −1 SD or any score outside the reference range on at least 1 of the cognitive tools as indicative for impairment of that particular cognitive domain.^23^

The presence of depression and anxiety symptoms, fatigue, and apathy was defined using well-established cutoffs of the Hospital Anxiety and Depression Scale (HADS) (>7 points for the depression subscale; >7 points for the anxiety subscale),^38^ the Fatigue Severity Scale (FSS) (≥4 points),^39^ and the Apathy Evaluation Scale–Self-report (AES-S) (≥34 points).^40^

Details about the testing of the cognitive and the emotional domains have previously been published.^23^

### Statistics

The Statistical Package for Social Sciences versions 27.0 and 28.0 were used for all statistical analyses.^a^ Descriptive statistics and table analyses were performed, and data are presented with means and SDs for continuous variables and with proportions and percentages for categorical variables. The rating scale scores are represented with median and range where the data distribution was skewed. To compare the baseline and the follow-up characteristics of the participants who returned to full-time work and those who did not, we performed chi-square (χ^2^) test for the categorical and independent samples *t* test for continuous variables.

Logistic regression analyses were performed separately to identify possible factors associated with the dependent variable of not returning to full-time work 12 months after a mild stroke. First, unadjusted regression analyses were performed using baseline patients’ characteristics, stroke characteristics such as topography, TOAST classification, functional level expressed as NIHSS, Barthel Index score at discharge from hospital, treatment with intravenous thrombolysis and thrombectomy, mRS score at discharge and follow-up, presence of cognitive impairment, and the scores on the anxiety, depression, fatigue, and apathy scales at 12-month follow-up as independent variables in unadjusted regression analyses.

Age, sex, and variables associated with the outcome with a *P* value <.1 in unadjusted analyses were then entered into multivariate hierarchical logistic regression models. In model 1 potential explanatory variables were baseline characteristics, that is, age, sex, years of education, diabetes mellitus, and NIHSS score at admission and discharge. The mRS score at follow-up and the scores of the anxiety, depression, and fatigue scales at 12-month follow-up were entered in model 2. The factors that remained significant from the hierarchical logistic models were considered to be the independent predictors of the outcome. Regarding the use of the anxiety, depression, fatigue, and apathy scales, we decided not to dichotomize these variables but to use the raw scores in order not to lose information and statistical power.^41^ Results are presented as odds ratio with 95% confidence interval. A *P* value <.05 was considered as the limit for statistical significance.

### Ethics

The Regional Committee South East for Medical and Health research ethics (register no. 2014/1268) and the Oslo University Hospital's Data Protection Authority approved the study. Before inclusion, written informed consent was given by all patients.

## Results

In the main study, we included 127 patients, with follow-up data on 117 of them. This substudy comprises 84 patients (17 female [20%]), with mean age 52.4±10.8 years who worked full-time at stroke onset. A total of 22 patients (25%) were treated with intravenous thrombolysis. Four patients (5%) were treated with mechanical thrombectomy, all combined with intravenous thrombolysis. Demographic and clinical baseline characteristics are presented in Table 1*.*

**Table 1:** Patient characteristics at baseline

Variables	Total Sample (N=84)	Male (n=67)	Female (n=17)
Demographics			
Male, n (%)	67 (80)		
Age (y), mean ± SD	52.4±10.8	54.0±10.4	46.0±10.5
Age (y), median (range)	55 (30-70)	56 (32-70)	44 (30-67)
Age group 30-45 y, n (%)	26 (31)	16 (24)	10 (59)
Age group >45 y, n (%)	58 (69)	51 (76)	7 (41)
Education in years, mean ± SD	15.6±3.4	15.6±3.4	15.7±3.9
Living alone, n (%)	22 (26)	18 (27)	4 (24)
Risk factors, n (%)			
Hypertension	39 (46)	36 (54)	3 (18)
Hyperlipidemia	61 (73)	54 (81)	7 (41)
APOE-ε4 allele	24 (30)	20 (31)	4 (24)
Coronary disease	8 (10)	8 (12)	0 (0)
Atrial fibrillation	6 (7)	5 (8)	1 (6)
Cigarette smoking	23 (27)	19 (28)	4 (24)
Diabetes mellitus	11 (13)	10 (15)	1 (6)
BMI, mean ± SD	26.9±4.2	27.3±4.2	25.5±3.8
Given thrombolysis, n (%)	22 (26)	12 (18)	10 (59)
TOAST classification, n (%)			
Large vessel disease	16 (19)	13 (19)	3 (18)
Cardiac embolic disease	25 (30)	19 (28)	6 (35)
Small vessel disease	20 (24)	18 (27)	2 (12)
Stroke of unknown/other etiology	23 (27)	17 (25)	6 (35)
Topography, MRI/CT findings, n (%)			
Right hemisphere	30 (36)	26 (39)	4 (24)
Left hemisphere	21 (25)	15 (22)	6 (35)
Cerebellum/brainstem	15 (18)	11 (16)	4 (24)
Multiple brain regions	14 (17)	12 (18)	2 (12)
No acute infarcts	4 (5)	3 (5)	1 (6)
Assessments			
NIHSS at admission, median (range)	1 (0-22)	1 (0-22)	2 (0-8)
NIHSS at discharge, median (range)	0 (0-3)	0 (0-3)	0 (0-3)
mRS score at discharge, median (range)	1 (0-4)	1 (0-4)	1 (0-3)
mRS score 0-1 at discharge, n (%)	67 (80)	54 (81)	13 (77)
BI at discharge, mean ± SD	19.8±1.1	19.7±1.3	19.9±0.3
BI at discharge, median (range)	20 (11-20)	20 (11-20)	20 (19-20)
Cognitive assessments			
IQCODE, mean ± SD	3.0±0.1	3.2±0.1	3.0±0.0

NOTE. Cigarette smoking, (≥1 cigarette/d); coronary artery disease: myocardial infarction, angina pectoris; hyperlipidemia: cholesterol >5 mmol/L or low-density lipoprotein cholesterol >3 mmol/L.

Abbreviations: APOE-ε4, apolipoprotein E-epsilon 4; BI, Barthel Index; BMI, body mass index (calculated as weight in kilograms divided by height in meters squared); CT, computed tomography; IQCODE, Informant Questionnaire on Cognitive Decline in the Elderly; MRI, magnetic resonance imaging; TOAST, Trial of Org 10172 in Acute Stroke Treatment.

At 12-month follow-up, 78 patients (93%) attended. During the 1-year poststroke period, 1 patient died, 3 had a recurrent stroke, and 2 withdrew from the follow-up. A total of 63 patients (81%) had returned to work, 47 (60 %) of them to full-time work. Table 2 shows the distribution of the employment status as well as cognitive and emotional impairments at follow-up for all patients included in the study. Tables 3 and 4 show the baseline and follow-up characteristics of patients who returned to full-time work 1 year after stroke and those who did not.

**Table 2:** Patient characteristics at 12-month follow-up

Variable	Total Sample N=78	Male n=62	Female n=16
Demographics			
Male, n (%)	62 (79)		
Employment status, full time, n (%)	47 (60)	42 (68)	5 (31)
Employment status, part time, n (%)	16 (21)	10 (16)	6 (38)
Employment status, full AND part time, n (%)	63 (81)	52 (84)	11 (69)
Full-time work after thrombolysis (n=21), n (%)	11 (52)	9 (75)	2 (22)
Assessments			
mRS score, median (range)	1 (0-3)	1 (0-3)	1 (0-3)
mRS score 0-1, n (%)	59 (76)	50 (81)	9 (56)
Barthel Index, median (range)	20 (20-20)	20 (20-20)	20 (20-20)
Impairments			
Cognitive impairments, n (%)	49 (63)	41 (66)	8 (50)
Emotional assessments			
HADS-Anxiety Scale, median (range)	4 (0-15)	4 (0-15)	5 (0-11)
HADS-Depression Scale, median (range)	2 (0-19)	1.50 (0-19)	2 (0-7)
FSS, median (range)	2.66 (1.00-6.77)	2.44 (1.00-6.77)	4.44 (1.00-6.22)
AES-S, median (range)	27 (18-52)	27 (18-52)	26 (18-38)

NOTE. Cognitive impairment, a score outside the reference range on at least 1 of the used cognitive tests.^20^

Abbreviations: AES-S, Apathy Evaluation Scale–Self-report; FSS, Fatigue Severity Scale; HADS, Hospital Anxiety and Depression Scale.

**Table 3:** Baseline characteristics of patients who returned to FTW 1 year after stroke and those who did not

Variable	FTW n=47	NFTW n=31	*P* Value*
Demographics			
Male, n (%)	42 (89)	20 (65)	
Female, n (%)	5 (11)	17 (35)	.01†
Age (y), mean ± SD	52.1±11.1	51.8±10.3	.68
Age (y), median (range)	53 (31-70)	55 (30-67)	
Age group 30-45 y	14 (30)	11 (35)	
Age group >45 y	33 (70)	20 (65)	.46
Education in years, mean ± SD	16.4±3.4	15.0±3.4	.04†
Living alone, n (%)	11 (23)	11 (35)	.38
Risk factors, n (%)			
Hypertension	22 (47)	14 (45)	.96
Hyperlipidemia	33 (70)	24 (77)	.66
APOE-ε4 allele	14 (31)	8 (26)	.51
Coronary disease	2 (4)	2 (7)	> 1.00
Atrial fibrillation	3 (6)	2 (7)	> .99
Cigarette smoking	10 (21)	11 (35)	.45
Diabetes mellitus	3 (6)	7 (23)	.08
BMI, mean ± SD	27.5±4.2	26.5±4.0	.37
Given thrombolysis	11 (23)	10 (32)	.42
TOAST classification			
Large vessel disease	9 (19)	6 (19)	.98
Cardiac embolic disease	13 (28)	10 (32)	.66
Small vessel disease	12 (26)	7 (23)	.77
Stroke of unknown/other etiology	13 (28)	8 (26)	.86
Topography, MRI/CT findings, n (%)			
Right hemisphere	18 (38)	9 (29)	.62
Left hemisphere	10 (21)	9 (29)	.62
Cerebellum/brainstem	9 (19)	6 (19)	.98
Multiple brain regions	7 (15)	6 (19)	.64
No acute infarcts	3 (6)	1 (3)	.65
Assessments			
NIHSS at admission, median (range)	0 (0-15)	2 (0-22)	.04†
NIHSS at discharge, median (range)	0 (0-3)	0 (0-3)	.01†
mRS score at discharge, median (range)	1 (0-4)	1 (0-4)	.16
mRS score 0-1 at discharge, n (%)	40 (85)	23 (74)	.08
BI at discharge, mean ± SD	19.9±0.4	19.5±1.8	.11
BI at discharge, median (range)	20 (18-20)	20 (11-20)	
Cognitive assessments			
IQCODE, mean ± SD	3.0±0.1	3.0±0.1	.24

NOTE. Cigarette smoking, (≥1 cigarette/d); coronary artery disease: myocardial infarction, angina pectoris; hyperlipidemia: cholesterol >5 mmol/L or low-density lipoprotein cholesterol >3 mmol/L.

Abbreviations: APOE-ε4, apolipoprotein E-epsilon 4; BI, Barthel Index; BMI, body mass index (calculated as weight in kilograms divided by height in meters squared); CT, computed tomography; FTW, returned to full-time work; IQCODE, Informant Questionnaire on Cognitive Decline in the Elderly; MRI, magnetic resonance imaging; NFTW, did not return to full-time work; TOAST, Trial of Org 10172 in Acute Stroke Treatment.

⁎ *P* value: χ^2^ test is performed for categorical variables; independent samples *t* test is performed for continuous variables.

† Statistical differences (*P*<.05).

**Table 4:** Follow-up characteristics of patients who returned to FTW 1 year after stroke and those who did not

Variable	FTW n=47	NFTW n=31	*P* Value*
Assessments			
mRS score, median (range)	1 (0-2)	1.5 (0-3)	<.01†
mRS score 0-1, n (%)	44 (94)	15 (48)	<.01†
Barthel Index, median (range)	20 (20-20)	20 (20-20)	
Impairments			
Cognitive impairments, n (%)	28 (60)	21 (68)	.25
Emotional assessments			
HADS-Anxiety Scale, median (range)	3 (0-10)	5.50 (0-15)	<.01†
HADS-Depression Scale, median (range)	1 (0-10)	3 (0-19)	<.01†
FSS, median (range)	2.00 (1.00-5.77)	4.11 (1.00-6.77)	<.01†
AES-S, median (range)	27 (18-52)	30.50 (18-50)	.22

NOTE. Cognitive impairment: a score outside the reference range on at least 1 of the used cognitive tests.^20^

Abbreviations: AES-S, Apathy Evaluation Scale–Self-report; FSS, Fatigue Severity Scale; FTW, returned to full-time work; HADS, Hospital Anxiety and Depression Scale; NFTW, did not return to full-time work.

⁎ *P* value: χ^2^ test is performed for categorical variables; independent samples *t* test is performed for continuous variables.

† Statistical differences (*P*<.05).

### Factors determining not returning to full-time work 12 months after a mild ischemic stroke

In the adjusted regression models, a low functional level (mRS score >1) at follow-up was significantly associated with not returning to full-time work, and diabetes mellitus showed a borderline (*P*=.052) significant association. The relationship between not returning to full-time work and female sex, NIHSS score at discharge, and a higher score on the anxiety, depression, and fatigue scales were deflated after adjusting for relevant covariates (Table 5).

**Table 5:** Predictors of not returning to full-time work 12 months after a mild ischemic stroke

	Unadjusted		Adjusted/Model 1		Adjusted/Model 2	
Variable	OR (95% CI)	*P* Value	OR (95% CI)	*P* Value	OR (95% CI)	*P* Value
Age at baseline	1.00 (0.96-1.04)	.91	1.10 (0.96-1.07)	.76	1.00 (0.94-1.07)	.95
Female sex	4.62 (1.41-15.09)	.01*	7.13 (1.71-29.71)	.007	3.43 (0.55-21.22)	.19
Living alone	1.80 (0.66-4.89)	.25				
Years of education	0.88 (0.76-1.00)	.08	0.84 (0.70-1.01)	.06	0.86 (0.69-1.07)	.19
BMI at admission	0.94 (0.83-1.06)	.28				
Hyperlipidemia	1.46 (0.51-4.15)	.48				
Diabetes mellitus	4.28 (1.01-18.07)	.05	6.33 (1.19-33.63)	.03	10.56 (0.98-113.47)	.052
Hypertension	0.94 (0.38-2.33)	.89				
Myocardial infarction	1.55 (0.21-11.64)	.67				
Atrial fibrillation	1.01 (0.16-6.43)	.99				
Presence of any APOE-ε4 allele	0.77 (0.28-2.14)	.62				
Treatment with thrombolysis	1.56 (0.57-4.29)	.39				
Treatment with thrombectomy	4.93 (0.49-49.72)	.18				
Stroke location (no infarcts at MRI/CT)	0.49 (0.05-4.93)	.54				
Stroke location (right hemisphere)	0.66 (0.25-1.74)	.40				
Stroke location (left hemisphere)	1.51 (0.53-4.30)	.44				
Stroke location (cerebellum/brainstem)	1.01 (0.32-3.20)	.98				
Stroke location (multiple infarctions)	1.37 (0.41-4.55)	.61				
TOAST (large vessel disease)	1.01 (0.32-3.20)	.98				
TOAST (cardiac embolic)	1.25 (0.46-3.34)	.66				
TOAST (small vessel disease)	0.85 (0.29-2.47)	.77				
TOAST (underdetermined/other etiology)	0.91 (0.33-2.54)	.86				
NIHSS at admission	1.13 (0.99-1.30	.07	1.10 (0.95-1.27)	.20	1.16 (0.96-1.40)	.13
NIHSS at discharge	1.59 (1.00-2.55)	.05	1.37 (0.75-2.50)	.31	1.11 (0.53-2.33)	.78
Barthel Index score at discharge	0.66 (0.33-1.32)	.24				
mRS score >1 at discharge	1.99 (0.64-6.19)	.24				
HADS-Anxiety Scale score	1.24 (1.07-1.45)	<.01*			1.20 (0.88-1.63)	.26
HADS-Depression Scale score	1.27 (1.07-1.49)	<.01*			0.98 (0.72-1.35)	.91
FSS score	2.01 (1.41-2.85)	<.01*			1.43 (0.77-2.63)	.26
AES-S score	1.05 (0.99-1.11)	.11				
Cognitive impairment	1.43 (0.55-3.69)	.47				
mRS score >1 at follow-up	15.64 (3.99-61.28)	<.01*			12.44 (2.37-65.43)	.003*

NOTE. Cognitive impairment: a score outside the reference range on at least 1 of the used cognitive tests.^20^

Abbreviations: AES-S, Apathy Evaluation Scale–Self-report; APOE-ε4, apolipoprotein E-epsilon 4; BMI, body mass index (calculated as weight in kilograms divided by height in meters squared); CI, confidence interval; CT, computed tomography; FSS, Fatigue Severity Scale; HADS, Hospital Anxiety and Depression Scale; MRI, magnetic resonance imaging; OR, odds ratio; TOAST, Trial of Org 10172 in Acute Stroke Treatment.

⁎ Statistical differences (*P*<.05).

## Discussion

We included patients who worked full time at onset of a first-ever mild ischemic stroke. At 12-month follow-up, 4 in 5 patients were back to any kind of employment, either full time or part time, of whom 3 in 4 worked full time. Reduced functional status (mRS score >1) at follow-up and having diabetes mellitus were associated with not returning to full-time work after 12 months. Female sex, NIHSS score at discharge, and a high score on anxiety, depression, and fatigue scales were also associated with not returning to full-time work, although these associations were only seen in the unadjusted regression models.

It has previously been reported that approximately 50% of patients return to work 1 year after stroke, and approximately 60% return 2 years after stroke.^4^^,^^19^^,^^42^ A greater percentage of our patients returned to paid work. This may not fully be explained by the young age in our sample because other studies^4^ also report on an average age between 37 and 55 years. Tables 1 and 3 show that the percentage of patients 45 years or younger (14/26 patients [54%]) returning to full-time work is nearly the same as the percentage of patients older than 45 years (33/58 [57%]). Generally, young working patients wish to return to their job and daily life as soon as possible after stroke.^19^ Older employees may be unable to work because of age-related changes or other health issues or prefer not to work as much as they did before stroke onset.^43^ On the other hand, it is possible that employers as well as the rehabilitation system focus more on younger than older working patients.

We found that low functional status at 12-month follow-up, operationalized as an mRS score >1, was an independent associative factor of not returning to full-time work. This may be because high mRS score expresses a higher dependence in daily life, which hinders patients from returning to work. An Indian study^44^ showed that functional disability after stroke and the type of job can determine return to work rather than psychosocial factors such as depression and anxiety. In addition, functional level at discharge^19^ , 1 month^45^, and 3 months^46^ post stroke can affect work participation.

In patients with stroke, diabetes mellitus has been shown to be both a risk factor for cognitive impairment^47^ and reduced ability to return to work.^6^^,^^8^^,^^19^^,^^43^ We found that diabetes is borderline significantly associated with not returning to full-time work, but we did not find any significant relationship between cognitive impairment and unemployment 12 months after stroke. Anxiety, depressive symptoms, and fatigue were related to not returning to full-time work but only in the unadjusted analyses. Such invisible emotional impairments have previously been described as factors affecting both quality of life^48^, work, and social participation^10^^,^^42^^,^^46^^,^^49^ after stroke.

High education (especially at university level) is related to high socioeconomical status, private insurance, and high income, and these factors are associated with a higher probability of returning to work up to 4 years after stroke onset^19^^,^^45^^,^^50^. In our study, we did not show that educational level was associated with returning to full-time work. However, other socioeconomic factors, such as patients’ income and profession were not recorded, and so the importance of these factors on returning to full-time work remains unclear. According to data from several studies^19^^,^^43^^,^^45^^,^^50^, men seem to return to work sooner than women. One explanation might be that women are less likely to have a favorable outcome^51^ and a good health-related quality of life^48^ after stroke than men. In our study there were fewer women (56%) with a favorable functional outcome (mRS score 0-1) than men (79%).

Some studies have identified a relationship between living alone and not returning to any kind of paid work.^44^^,^^52^ Possible explanations for these findings could be that people who live alone after stroke may have more anxiety and less support and encouragement from a partner in their daily life^52^. We did not, however, find that living alone at stroke onset predicts not returning to full-time work.

The effect of reperfusion therapy on the outcome after stroke is widely investigated during the last years^12^, but there are only a few studies^15^^,^^16^ that describe that treatment of acute ischemic stroke with intravenous thrombolysis may be a positive predictor of returning to full-time work after stroke. Approximately 50% of our patients treated with intravenous thrombolysis returned to full-time work 1 year later, but the association between thrombolysis and return to work was not statistically significant. This may be because of low power related to few patients or the study sample with patients who had a high functional level expressed as mRS score ≤1 at discharge (74%).

### Study limitations and strengths

One limitation was that only 1 in 5 participants were female. The large number of male patients younger than 70 years, however, is in line with stroke epidemiology.^53^

One of our inclusion criteria was that patients were previously cognitively intact. To reduce the possibility of underreported prestroke cognitive decline in some patients, the information about cognitive health was obtained through interviews with patients, their next of kin, and their family physicians and by reviewing patients’ medical records. The dependents of all patients filled in the Informant Questionnaire on Cognitive Decline in the Elderly.^25^

Strengths of the study are the clear diagnosis and classification of a first-ever mild stroke. We used validated and widely used cognitive and emotional instruments to evaluate several cognitive and emotional domains. The tests were performed by either a physician, occupational therapist, or nurse. We did not perform any intrarater reliability tests of the tools used in our study, and this may represent a limitation.

Almost all patients aged 18-70 years admitted to the 2 stroke units during the inclusion period and who had been evaluated as eligible for inclusion, wished to participate. Only 3 of the included patients withdrew their consent before follow-up. Consequently, attrition bias was low.

## Conclusions

Younger patients can have difficulties with returning to paid work even after a mild stroke without visible neurologic deficits. Low functional level that persists 12 months after stroke may be related to not returning to full-time work. Patients with diabetes mellitus, female patients, and patients with a higher score on fatigue, anxiety, and depression scales may also be at risk of not returning to full-time job post stroke. We suggest that in future studies and clinical practice, working patients should be offered a poststroke follow-up with particular focus on identifying factors that can affect participation in work and social life.

## Supplier

a. SPSS versions 27.0 and 28.0; IBM.

## Appendix Supplementary materials

Image, application 1
