# Supplementary material for: Factors Determining Not Returning to Full-Time Work 12 Months After Mild Ischemic Stroke
Source: Arch Rehabil Res Clin Transl. 2022 Nov 12;5(1):100245. doi: 10.1016/j.arrct.2022.100245 (PMC10036226; doi:10.1016/j.arrct.2022.100245)
Supplement: Supplementary file 1 [file mmc1.docx]

| **Table I.** **Cognitive and emotional functioning of all patients 12 months after mid ischemic stroke.** | | | |
| --- | --- | --- | --- |
| Variables | Total sample  N=78 | Male  n=62 | Female  n=16 |
| Impairments |  |  |  |
| Cognitive impairments | 49 (63) | 41 (66) | 8 (50) |
| MMSE-NR2 score < 27/30 | 4 (5) | 2 (3) | 2 (13) |
| Clock drawing score <4/5 | 2 (3) | 1 (2) | 1 (6) |
| TMT A, time > 60 sec | 0 (0) | 0 (0) | 0 (0) |
| TMT B, time >120 sec | 6 (8) | 5 (8) | 1 (7) |
| COWA, letter fluency ≤ -1 SD | 29 (38) | 27 (44) | 2 (13) |
| ROCF, copy ≤-1 SD | 4 (5) | 3 (5) | 1 (7) |
| ROCF, delayed recall ≤-1 SD | 19 (25) | 16 (26) | 3 (20) |
| CVLT-II, total learning ≤-1 SD | 5 (7) | 3 (5) | 2 (13) |
| CVLT-II, short delay free recall ≤ -1SD | 10 (13) | 7 (12) | 3 (19) |
| CVLT-II, long delay free recall ≤ -1SD | 7 (9) | 5 (8) | 2 (13) |
| Emotional assessments |  |  |  |
| HADS-Anxiety Scale, median (range) | 4 (0-15) | 4 (0-15) | 5 (0-11) |
| HADS-Anxiety Scale, >7 points | 13 (17) | 8 (13) | 5 (31) |
| HADS-Depression Scale, median (range) | 2 (0-19) | 1.50 (0-19) | 2 (0-7) |
| HADS-Depression Scale, >7 points | 8 (10) | 7 (11) | 1 (6) |
| FSS, median (range) | 2.66 (1.00-6.77) | 2.44 (1.00-6.77) | 4.44 (1.00-6.22) |
| FSS, ≥ 4 points | 26 (33) | 16 (26) | 10 (63) |
| AES-S, median (range) | 27 (18-52) | 27 (18-52) | 26 (18-38) |
| AES-S, ≥ 34 points | 20 (26) | 15 (24) | 5 (33) |
| NOTES: n (%) for categorical data, unless otherwise specified.  Cognitive impairment, a score outside the reference range on at least one of the used cognitive tests [20]; MMSE-NR2, Mini Mental Status Examination Norwegian Revision 2; TMT A, Trail Making Test A; TMT B, Trail Making Test B; COWA, Controlled Oral Word Association; ROCF, Rey-Osterrieth Complex Figure; CVLT II, California Verbal Learning Test-II; HADS, Hospital Anxiety and Depression Scale; FSS, Fatigue Severity Scale; AES-S, Apathy Evaluation Scale-Self Report | | | |

| **Table II. Cognitive and emotional functioning of patients who returned to full-time work (FTW) and those who did not (NFTW) 12 months after mild ischemic stroke.** | | |
| --- | --- | --- |
| Variables | FTW  N=47 | NFTW  N=31 |
| Impairments |  |  |
| Cognitive impairments | 28 (60) | 21 (68) |
| MMSE-NR2 score < 27/30 | 0 (0) | 4 (13) |
| Clock drawing score <4/5 | 0 (0) | 2 (7) |
| TMT A, time > 60 sec | 0 (0) | 0 (0) |
| TMT B, time >120 sec | 2 (4) | 4 (13) |
| COWA, letter fluency ≤ -1 SD | 19 (41) | 10 (33) |
| ROCF, copy ≤-1 SD | 0 (0) | 4 (13) |
| ROCF, delayed recall ≤-1 SD | 10 (21) | 9 (30) |
| CVLT-II, total learning ≤-1 SD | 2 (4) | 3 (10) |
| CVLT-II, short delay free recall ≤ -1SD | 3 (7) | 7 (23) |
| CVLT-II, long delay free recall ≤ -1SD | 3 (7) | 4 (13) |
| Emotional assessments |  |  |
| HADS-Anxiety Scale, median (range) | 3 (0-10) | 5.50 (0-15) |
| HADS-Anxiety Scale, >7 points | 4 (9) | 9 (29) |
| HADS-Depression Scale, median (range) | 1 (0-10) | 3 (0-19) |
| HADS-Depression Scale, >7 points | 2 (4) | 6 (19) |
| FSS, median (range) | 2.00 (1.00-5.77) | 4.11 (1.00-6.77) |
| FSS, ≥ 4 points | 9 (19) | 17 (55) |
| AES-S, median (range) | 27 (18-52) | 30.50 (18-50 |
| AES-S, ≥ 34 points | 9 (19) | 11 (37) |
| NOTES: n (%) for categorical data, unless otherwise specified.  Cognitive impairment, a score outside the reference range on at least one of the used cognitive tests [20]; MMSE-NR2, Mini Mental Status Examination Norwegian Revision 2; TMT A, Trail Making Test A; TMT B, Trail Making Test B; COWA, Controlled Oral Word Association; ROCF, Rey-Osterrieth Complex Figure; CVLT II, California Verbal Learning Test-II; HADS, Hospital Anxiety and Depression Scale; FSS, Fatigue Severity Scale; AES-S, Apathy Evaluation Scale-Self Report | | |
